# Fractured Patella Treated by Wiring

**Published:** 1888-10

**Authors:** J. J. Buchanan

**Affiliations:** Pittsburg, Pa.


### FRACTURED PATELLA TREATED BY WIRING.*

BY J. J. BUCHANAN, M. D., PITTSBURG, PA.

The patient is a German laborer, and his fracture was the result of direct violence, caused by the stroke of a three hundred-pound box which fell against his knee. He stated that the accident happened in the middle of the day of June 30th. He continued to do his laboring work till evening, but on the following day found that he was unable to stand on the limb. I suppose that the blow broke the bone, but the capsule held together till evening. When he was brought to the hospital, five days afterward, the joint was considerably distended and the fracture easily recognized, but the lower fragment seemed to be very small. He was informed of the probable result by the use of external appliances, and the advantages as well as the risks attending the method by suture. With a full understanding of the circumstances, he demanded the treatment which would give him the most useful limb, even though at some slight risk to his life. I accordingly operated on the eleventh day after the injury.

The most scrupulous precautions against sepsis were taken.

*A paper read at Allegheny County Medical Society.

Instruments and appliances were put through the same course of preparation as for laparotomy. Continuous irrigation with 1-2500 sublimate solution was employed, and the transverse incision was made to the full extent of the rent in the capsule. The lower fragment was not larger than a chestnut. The capsule was much lacerated, and a number of narrow shreds hung into the joint; the joint contained a great deal of clotted blood and bloody fluid. The joint was thoroughly washed out and all loose pieces and ragged ends and edges of capsule were cut away with scissors. The fractured surfaces were refreshed by the vigorous use of a curette.

A single hole was drilled through each fragment, the drill entering about three-eighths of an inch from the line of fracture, and emerging at the cartilaginous border of the fractured surface. As a motive-power for the drill, I used the dental engine, which was kindly supplied and manipulated for me by Dr. Charles Phillips, a dentist of this city.

A silver wire of No. 24 gauge was passed. An incision was made into the lower part of the joint on the outside of the limb and a rubber drain inserted, the inner extremity barely entering the joint. The silver wire was then twisted firmly, which brought the fragments into place, and the ends of the wire were turned down between the edges of the apposed fragments. The capsule was closely united over the whole length of the rupture with the continuous catgut suture.

Interrupted silk-worm-gut stitches were used for the soft parts down to the capsule. Sublimated dressings and a posterior splint completed the work. At the expiration of the third day the drain was exposed and withdrawn. The primary dressing was removed at the end of a week, when the wound of the soft parts was found to be soundly healed and the skin stitches were all taken out. The progress of the case was aseptic, and, of course, absolutely devoid of pain and discomfort.

At the end of four weeks the patient was allowed out of bed, and at the end of five and a half weeks all dressings were removed and he was allowed to walk upon the limb with the aid of crutches. At the end of six and a half weeks he was permitted to rely on a cane without any support to the limb. When I last examined him, four or five days ago, palpation of the patella gave no evidence of its ever having been fractured. The range of motion is not yet great, but is rapidly increasing and will, I doubt not, be completely restored.

There is no question that the treatment of fractured patella by external retentive apparatus is extremely unsatisfactory. An occasional case of close ligamentous union encourages the surgeon, but the great majority of cases have a half inch or more of separation which gradually increases; a large proportion have refracture or rupture of the ligament and almost all have limbs of greatly impaired usefulness.

This operation, when it succeeds, as it usually does, is said to leave the patient with bony union and with a freely movable joint. It certainly is the most speedy and least troublesome of all methods of treatment. I myself think it is destined to be the treatment of the future. As our methods of securing asepsis of operative wounds become more certain and our skill in applying them increases, so will the patella suture become better established. In the present condition of the science the mortality of this operation is slight, but it still exists. I think it will be reduced practically to zero. As things now are I think the advisability of the operation in any particular case should depend upon the wishes of the patient and the skill of the operator in securing asepsis.

If the patient is unwilling, or his attendant lacks the technical skill for rigid antisepsis, the operation should not be thought of. On these points I can do no better than to quote the words of Dr. Frank W. Rockwell, of Brooklyn: "Finally, I believe that so long as this form of fracture is treated by the ordinary methods employed, just so long will the present unsatisfactory results continue to obtain, and I believe it to be the duty of the surgeon, in any given case, to at least give his patient the benefit of deciding for himself whether he will have wiring done or not, and, in event of his selecting the operation, to do it at the earliest proper time, if capable of performing a thoroughly aseptic operation, since I believe that by so doing he will obtain the best results in the largest number of cases."

To the same effect has Dr. Lewis S. Pilcher, also of Brooklyn, expressed himself:

"The whole principle of exposing the patella and refreshing the fragments and bringing them together is the outgrowth of the antiseptic principle, and to a very considerable extent it may be considered one of the most difficult achievements of antiseptic work. Now, it seems to me that, in expressing an opinion upon the justifiability of an operation of this kind, we ought to qualify it somewhat in this way: That a surgeon who has become a master of the practice of antisepsis, as well as the principles, and who is able to control with certainty the conditions which surround his patient, would be justified in opening the knee-joint in a recent case of fracture of the patella and bringing the fragments together; but I doubt very much whether, excepting under such circumstances, it would be justifiable."

